# The impact of patient support and health education on diabetes management and glycemic control

**DOI:** 10.25122/jml-2024-0290

**Published:** 2024-10

**Authors:** Meshari Nawar Alotaibi, Aminah saeed Almutairi, Fahad Abdulmajeed Alkhayal, Sulaiman Mohammed Alqahtani, Fatimah nasser Alshehri, Mostafa Kofi

**Affiliations:** 1 Department of Family and Community Medicine, Prince Sultan Military Medical City (PSMMC), Alsalam Center, Riyadh, Saudi Arabia; 2 Department of Family and Community Medicine, Prince Sultan Military Medical City (PSMMC), Riyadh, Saudi Arabia

**Keywords:** Type 2 diabetes mellitus (T2DM), educational programs, glycemic control, patient support, diabetes management, T2DM, Type 2 Diabetes Mellitus, DSMES, Diabetes Self-Management Education and Support, HbA1c, Glycated Hemoglobin, SEDM, Structured Education in Diabetes Management, ROB v2, Risk of Bias Version 2

## Abstract

Diabetes mellitus is a chronic and complex medical condition that can lead to significant morbidity and mortality. Maintaining an adequate blood glucose level is important for patients with diabetes, and to improve glycemic control, patients need proper support and health education, which are essential components of comprehensive diabetes care. We used a rigorous approach based on the PRISMA and Cochrane Handbook principles, specifically focusing on randomized controlled trials (RCTs) published in English from 2005 onwards. The statistical analysis was conducted using the RevMan software. Pooled risk ratios were calculated for dichotomous data, whereas mean differences were calculated for continuous data. Heterogeneity and publication bias were also evaluated. From an initial pool of 544 records, 368 studies were examined after eliminating duplicates. Ultimately, 24 studies were deemed suitable based on the inclusion criteria. These studies involved 2437 participants in the intervention group and 2305 people in the control group. The quality assessment indicated that 41.7% of the studies were categorized as low risk, 16.7% as high risk, and 41.7% had certain concerns regarding bias. The analyses revealed noteworthy decreases in HbA1c levels in the intervention group at several time points, particularly showing improvements after 3 months. Egger's regression indicated the presence of possible publication bias. The results emphasize the crucial impact of health education and mentorship interventions on enhancing glycemic control in individuals with type 2 diabetes. Interventions focused on empowering patients proved to be especially effective in enhancing diabetes management outcomes.

## INTRODUCTION

Type 2 diabetes mellitus (T2DM) is a complex medical condition characterized by chronic hyperglycemia. Over the past three decades, the prevalence of diabetes mellitus has quadrupled worldwide, ranking it as the ninth most common cause of death [1]. Currently, diabetes affects 1 in 11 people worldwide, with approximately 90% of cases being T2DM [1]. Key contributors to the global rise in T2DM include obesity, sedentary lifestyles, and the increased consumption of unhealthy diets [1]. Among patients with T2DM, cardiovascular complications are the leading cause of morbidity and mortality. Prevention strategies for T2DM include maintaining a healthy weight, eating a balanced diet, and exercising frequently [1].

Research studies have consistently shown a positive impact of patient support and health education on diabetes control. These studies emphasize the importance of education in improving knowledge, attitudes, and practices related to diabetes management, ultimately leading to better glycemic control and reduced complications [2]. Additionally, patient-centered care and self-care education have been found to optimize glycemic control and reduce the risk of cardiovascular disease in individuals with diabetes [3]. Furthermore, research indicates that diabetes education programs, such as diabetes self-management education and support (DSMES), enhance diabetes self-care practices. Individuals participating in diabetes education programs are more likely to adhere to recommended self-care and clinical-care practices, leading to better health outcomes. These programs help patients gain the necessary knowledge, skills, and support for effective diabetes self-management [4]. Effective management of T2DM goes beyond traditional approaches and has led to the exploration of empowerment-based interventions [5]. Empowerment can be viewed as a process in which individuals gain the knowledge, skills, attitudes, and self-awareness necessary to influence their behavior, thereby improving responsibility and autonomy and obtaining the power to make informed decisions [5]. These strategies have shown promise in improving glycemic control, self-efficacy, and diabetes knowledge among individuals with T2DM. For example, a meta-analysis by Chen and colleagues demonstrated that empowerment-based education significantly improved HbA1c levels, diabetes knowledge, and psychosocial self-efficacy compared to routine care. These findings highlight the potential of empowerment-based interventions to enhance T2DM management [5]. Our study aimed to assess the impact of patient support and health education on diabetes control by reviewing existing research on the topic.

## MATERIAL AND METHODS

This research evaluated the impact of patient support and health education programs on diabetes control. The investigation adhered to the guidelines outlined in the Preferred Reporting Items for Systematic Reviews and Meta-Analyses (PRISMA) guidelines [6] and the Cochrane Handbook of Systematic Reviews of Interventions [7]. The study protocol was registered on PROSPERO on 23 March 2024, under registration number CRD42024520732.

### Eligibility criteria

The PICOS framework was used to select randomized controlled trials (RCTs) for inclusion. The population included adults diagnosed with T2DM. The intervention involved patient support or health education programs, while the control group received conventional diabetes care. The primary outcome was the measurement of HbA1c levels, an indicator of diabetes control. Only full-text RCTs published in English from 2005 onwards were included. Other types of trial designs, research involving children or pregnant women, abstracts, protocols, non-English studies, review articles, comments, and case reports were not considered.

### Literature search strategy

A comprehensive search strategy was developed using specific keywords, Boolean operators (AND, OR), and MeSH terms. Keywords included 'patient support', 'patient education', 'health education', 'diabetic education', and 'diabetes management education', and terms associated with diabetes control such as 'glycemic control', 'blood glucose control', and 'HbA1c control'. The search was conducted across multiple databases, including PubMed, Cochrane, Web of Science, and Scopus, for studies published in English from 2005 onwards. During our assessment of reference lists, we specifically omitted research that was undertaken in non-English language, debates, conference papers, or dissertations that were not available. The initial author, MNA, conducted this stage by incorporating all pertinent studies without any restrictions or filters.

### Study selection

We conducted a systematic review by searching the databases PubMed, Cochrane, Web of Science, and Scopus for studies published from 2005 onwards. After the search, all identified articles were imported into EndNote to remove duplicates. Titles and abstracts were screened independently by MNA and SMA to assess eligibility. ASA and FNA performed full-text reviews to confirm the relevance of the studies. Any disagreements were resolved by the primary author.

### Data extraction

All team members contributed to this phase. Relevant data were systematically extracted and organized into Excel sheets. The general sheet included key information such as authors' names, study year, design, intervention and control types, medications used, and study outcomes. A separate sheet documented baseline characteristics like age, sex, and HbA1c for each study. The outcome sheet focused on HbA1c. Continuous data like age were summarized using mean and standard deviation, while dichotomous data such as gender were presented as event and total counts. Data from each study was arranged in columns for easy comparison across projects. Two researchers independently collected and tabulated the data. Additionally, every selected article was carefully reviewed by another author to avoid repetition or overlap of content.

### Quality assessment

Two independent authors, FAA and FNA, conducted quality assessments using the Cochrane risk-of-bias version 2 (ROB v2) tool for RCTs [8]. This method evaluates research based on five domains: randomization bias, intervention deviation bias, missing outcome data bias, outcome measurement bias, and selection bias in reported outcomes. The authors classified their assessments as low risk, high risk, or with particular concerns regarding bias.

### Statistical analysis

Statistical analyses were performed using Review Manager (RevMan) software version 5.4.1. Dichotomous data were analyzed using pooled risk ratio (RR), while continuous data were analyzed using mean difference. We utilized the random-effects model for analysis. Heterogeneity across studies was evaluated using Egger’s regression, and potential publication bias was assessed visually with a funnel plot.

### Assessment of heterogeneity

Heterogeneity among studies was evaluated using the I-squared (I^2^) statistic and the *P* value. The levels of heterogeneity were classified based on the I^2^ values as follows: insignificant (0–40%), moderate (30–60%), substantial (60–80%), and significant (80–100%). An analysis was deemed heterogeneous if the *P* value was below 0.05 or the I^2^ value exceeded 60%.

## RESULTS

**Figure 1:**
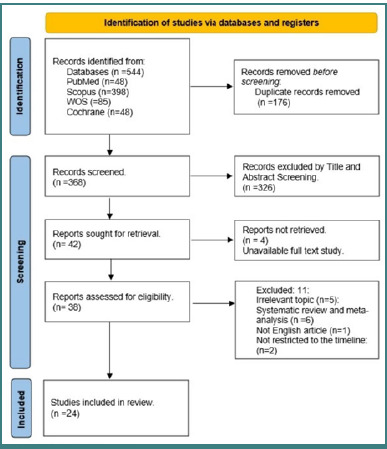
PRISMA flow diagram

A total of 544 results were retrieved from different databases, and 176 duplicates were removed. A total of 368 studies underwent title and abstract screening, which led to the exclusion of 326 studies. Out of the remaining 42 studies, 24 met our inclusion criteria, comprising 2,437 participants in the intervention group and 2,305 in the control group (Figure 1). The characteristics of the included studies are presented in Table 1 [9-32].

**Table 1:** Study characteristics

Study ID	Year	study design	Age mean (SD)		Male (event/total)		Type of intervention	Comparator	Results
			Intervention	Control	Intervention	Control			
Long *et al*. [9]	2020	RCT	Phase 1: 59.6(7.9) Phase 2: 62.3(6.9)	Phase 1: 60.6(7.4) Phase 2: 62.3(6.8)	Phase 1: 195/202 Phase2: 66/68	Phase 1: 146/154 Phase 2: 47/47	Received mentoring from peers with well-controlled diabetes whose diabetes was once in poor control	Usual care	HbA1c (6 months – 12 months), DDS
Riddell *et al*. [10]	2016	RCT	61.3 (9.3)	60.5 (8.7)	60/120	62/120	Peer support intervention	Routine care control	HbA1c (12 months), weight,
Shaya *et al*. [11]	2014	partial RCT	53.9	51.9	30/68	35/70	Diabetes education sessions	Control	HbA1c (3 months – 6 months), Blood glucose
Sugiyama *et al*. [12]	2015	RCT	63.7 (6.3)	63.3 (6.8)	81/285	69/258	DSME intervention (community-based diabetes self-management empowerment program)	Control	HbA1c (6 months), Social support score
Thanh *et al*. [13]	2021	RCT	61.5 (9.2)	62.9 (9.4)	81/182	83/182	Group education for T2DM knowledge, diet, exercise		HbA1c (3 months), Michigan total score, FBG
Torres *et al*. [14]	2018	RCT	62.1 (10.9)	62.5 (10.5)	67/231	78/239	Educational program	The control group was monitored individually	HbA1c (3 months – 6 months, 9 months)
Wichit *et al*. [15]	2017	RCT	61.3 (11.6)	62.1 (10.9)	17/70	21/70	A family-oriented program that included education classes, group discussions, a home visit, and a telephone follow-up	Routine care	HbA1c (5 weeks – 13 weeks), DMSES, PTES, SDSCA, PCS, MCS, DKQ
Yuan *et al*. [16]	2014	RCT	58.9 (8.4)	57.8 (8.2)	14/36	12/40	DSME program	Standard advice on medical nutrition therapy.	HbA1c (3 months), lipid profile, BG, SBP, DBP, DC, AC, Weight, BMI
Castillo-Hernandez *et al*. [17]	2021	RCT	59(9.4)	56(10.3)	2/29	0/29	(DSME and peer leader (PL) support): peer support and diabetes self-management education group (PSEG)	Conventional diabetes self-management education-only group	HbA1c (4 months – 8 months)
Lynch *et al*. [18]	2014	RCT	54.1 (33-77)	54.1 (33-77)	12/30	8/31	Lifestyle Improvement Through Food and Exercise (LIFE) Intervention	The control treatment consisted of two 3-hour self-management training classes	HbA1c (3 months), BMI, DBP, SBP, weight
Tang *et al*. [19]	2015	RCT	56.7 (11.5)	55.9 (11.3)	17/54	18/52	American community health worker		HbA1c (3 months – 9 months), BMI, DBP, SBP, weight, lipid profile
Heisler *et al*. [20]	2010	RCT	61.8 (6.1)	62.3 (6.6)	125/125	119/119	12 months of weekly group sessions and supplementary telephone support delivered by peer leaders	3-month DSME program with no follow-up peer support	HbA1c (6 months), DBP, SBP, Diabetic distress
Peimani *et al*. [21]	2017	RCT	59 (11.3)	58.8 (11.7)	53/100	51/100	Reciprocal peer support	Nurse care management (NCM)	HbA1c (6 months), BMI, Diabetes quality of life
Withidpanyawong *et al*. [22]	2019	RCT	60.53 (10.7)	58.13 (1.1)	24/88	23/92	Peer support	Control	HbA1c (9 months), BMI, lipid profile, DBP, SBP
Chen *et al*. [23]	2008	RCT	62 (10.1)	63.6 (8)	27/52	25/50	Family intervention	Control	HbA1c (9 months, 12 months), weight, FBG, Fructosamine
Santos *et al*. [24]	2017	RCT	59.2 (8.5)	57.5 (9.7)	34/93	38/111	Regular diabetes education	Special reminder pamphlet during the holidays	HbA1c (12 months), ESM, DES
Farsaei *et al*. [25]	2010	RCT	53.4 (9.8)	52.9 (8.5)	32/87	28/87	group education, home visit	Control group	HbA1c (3 months), FBG
Hosseini *et al*. [26]	2021	RCT	57.6 (8)	59.1 (7.1)	16/47	14/47	Educational sessions	Control	HbA1c (6 months), Knowledge, Attitude and behavior, FBG
Huang *et al*. [27]	2010	RCT	55.8 (8.2)	57.4 (7.5)	23/56	31/60	Educated by receiving ongoing instruction on the self-monitoring of glucose level	Control group of routine care practiced at their primary care	HbA1c (12 months), BMI, DBP, SBP, lipid profile, creatinine, uric acid level
Amendezo *et al*. [28]	2017	RCT	51.4 (10.9)	50.5 (11)	34/123	37/128	educational program based on the theory of planned behavior	Control group	HbA1c (12 months), FBG, DBP, SBP, weight
Castillo-Hernandez *et al*. [29]	2023	RCT	58 (14)	59 (11)	24/81	25/82	Registered Dietitian–Led Diabetes Management	Routine care Control group	HbA1c (12 months), DBP, SBP
Gomes *et al*. [30]	2017	RCT	47.1 (13.52)	-	18/108	-	lifestyle education program	Tertiary standard of care treatment	HbA1c (6 months-12 months), lipid profile, creatinine, urea
Gucciardi *et al*. [31]	2007	RCT	60.4 (7.92)	59 (12.1)		11/36	Peer-supported diabetes education program (receiving either culturally sensitive peer support on top of a diabetes self-management education group [PLG])	a diabetes self-management education group only	HbA1c (3 months), Attitude, nutrition adherence
Hermanns *et al*. [32]	2012	RCT	62 (8.7)	63.9 (7.8)	49/94	58/92	Family caregiver whom the patient recognized as a source of social support	control group	HbA1c (6 months), lipid profile, DBP, SBP

HbA1c, Hemoglobin A1c; DDS, Diabetic Distress Scale; FBG, Fasting Blood Glucose; DMSES, Diabetes Management Self-Efficacy Scale; PTES, Perceived Therapeutic Efficacy Scale; SDSCA, Summary of Diabetes Self Care Activities; PCS, Physical Component Summary; MCS, Mental Component Summary; DKQ, Diabetes Knowledge Questionnaire; SBP, Systolic Blood Pressure; DBP, Diastolic Blood Pressure; BG, Blood Glucose; ESM, Self-Care Questionnaire for Type 2 Diabetes Mellitus; DES, Empowerment Questionnaire for Type 2 Diabetes Mellitus

### Quality assessment

**Figure 2:**
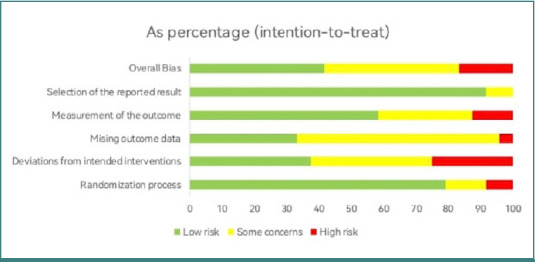
Summary of the quality assessment of the included RCT studies

**Figure 3:**
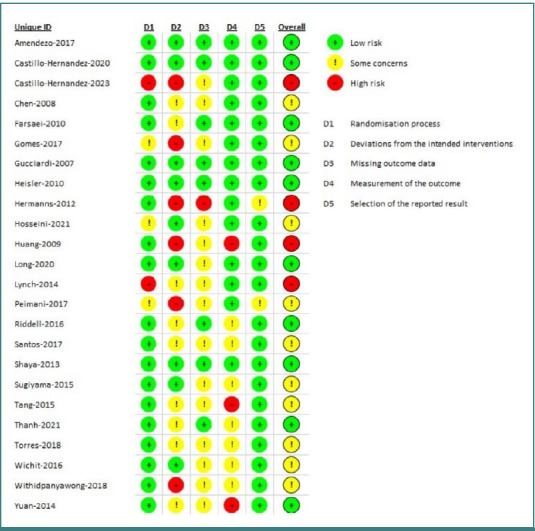
Quality assessment of the included RCTs by ROB v2 tool

Based on the ROB v2 assessment, the quality evaluation of the included RCTs indicated that 41.7% were classified as low risk, 16.7% as high risk, and 41.7% had some concerns regarding overall bias. The domains of randomization procedure and selection of presented results had a low risk of bias, with percentages of 79.2% and 91.7%, respectively. However, the percentage of missing outcome data in the domain was 62.5%, raising concerns about potential bias. The deviations from the targeted interventions domain had a high probability of bias, specifically 25% (Figure 2). Of the RCTs included in the review, 10 studies were classified as low risk, 10 had some concerns, and 4 were assessed as having a high risk of bias (Figure 3).

### Sensitivity and publication bias

**Figure 4:**
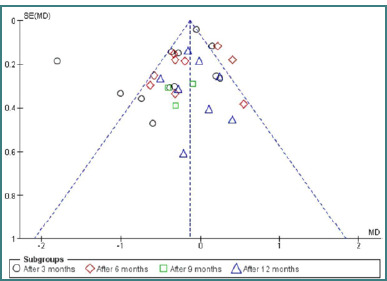
Funnel plot to assess the publication bias for the change in HbA1c

Egger’s regression intercept was used to detect publication bias of HbA1c [33]. The study size is shown on the vertical axis of the funnel plot as a function of the effect size on the horizontal axis. Scattered points on the plot represent the included studies. The asymmetry in our plot may be due to heterogeneity between studies, language bias, or reporting bias [34]. A subgroup analysis was conducted to investigate the asymmetry further (Figure 4).

### Efficacy outcomes

**Figure 5:**
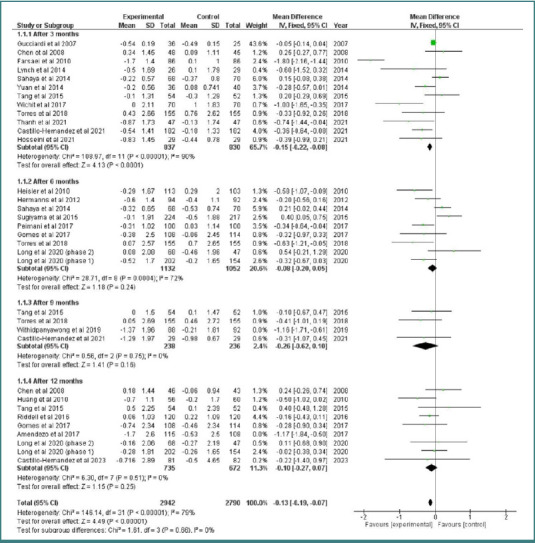
A forest plot for the change in the level of HbA1c over time

All included studies reported a change in the HbA1c level, significantly affected by the intervention [9-32]. A subgroup analysis was conducted to evaluate the impact of the intervention at various time points (Figure 5).

### Outcomes after 3 months

Out of the 24 included studies, 12 studies with a total of 837 participants reported changes in HbA1c levels after 3 months of intervention [11,13-19,23,25,26,31]. The overall effect size favored the intervention group over the control group after 3 months (MD = -0.15; 95% CI, -0.22 to -0.08; *P* < 0.0001). The pooled studies showed significant heterogeneity (Chi-square *P* < 0.0001, I^2^ = 90%).

### Outcomes after 6 months

A total of 1132 participants from eight studies were assessed after 6 months of intervention, compared to the control group of 1052 participants [9,11,12,14,20,21,30,32]. The effect size between the intervention and control groups was statistically insignificant (MD = -0.08; 95% CI, -0.2 to 0.05; *P* = 0.24). Our results were heterogeneous (Chi-square *P* = 0.004, I^2^ = 72%)

### Outcomes after 9 months

Four studies, with 238 participants, reported changes in HbA1c levels after 9 months. While the intervention group showed a greater reduction in HbA1c, the result was not statistically significant (MD = -0.26, 95% CI, -0.62 to 0.1; *P* = 0.16) [14,17,19,22]. Sensitivity analysis resolved the heterogeneity in these results (Chi-square *P* = 0.75, I^2^ = 0%).

### Outcomes after 12 months

Changes in HbA1c levels after 12 months were compared between the intervention and control groups in 8 studies involving 735 participants in the intervention group and 672 in the control group [9,10,19,23,27-30]. The overall effect between the groups was not statistically significant (MD = - 0.1; 95% CI, -0.27 to 0.07; *P* = 0.25). Heterogeneity was resolved through a leave-one-out test (Chi-square *P* = 0.51, I^2^ = 0%). The total effect size of the studies favored the intervention group over the control group (MD = -0.13; 95% CI, -0.19 to -0.07; *P* < 0.0001). Our results were heterogeneous even after subgrouping and conducting sensitivity analysis (Chi-square *P* < 0.0001, I^2^ = 79%) (Figure 5).

## DISCUSSION

Most of the included studies favored educational or mentoring methods in treating patients with T2DM. The results of our systematic review and meta-analysis showed a statistically significant effect of the education and mentoring method in decreasing the level of HbA1c over time compared to the control group. By conducting a subgroup analysis, we noticed that the intervention significantly affected patients after the first three months. The change in the level of HbA1c stopped at subsequent time points. The studies included in our meta-analysis showed significant heterogeneity, which was solved by conducting subgroup and sensitivity analysis. This heterogeneity may be attributed to the moderate quality of the included RCTs and the varying methods of mentoring used by the healthcare professionals involved. To account for these variations, we applied a random-effects model to fit the predicted outcomes better.

One study conducted by Santos *et al*. [24], which was included in our review, was not suitable for the meta-analysis due to the nature of the reported data. However, significant improvements in HbA1c levels were observed in participants receiving group education compared to those in the control and home visit groups [24]. Similarly, a randomized clinical trial by Maršić *et al*. found that education on medication adherence and side effects provided by pharmacotherapy experts improved drug adherence and enhanced clinical outcomes in patients with T2DM [35]. In a systematic review by Yorke *et al*., the integration of structured education in diabetes management (SEDM) into usual care significantly improved glucose control and hypoglycemia management in patients with T2DM, aligning with the results of our meta-analysis [36]. Many previously published meta-analyses have demonstrated that patient self-management education resulted in better outcomes in glucose control compared to usual care of patients by assessing the change in the level of HbA1c [37-39]. A recent meta-analysis conducted by Hildebrand and his colleagues focused on studies including Latino adults with T2DM and assessing the effect of DSME in reducing the level of HbA1c [40]. It showed that the HbA1c level was significantly affected by the intervention and showed preferable outcomes over the control group −0.240% (95% CI = −0.345 to 0.135, *P* < 0.001). Social media platforms have also emerged as a cost-effective tool for providing diabetic health education, empowering patients through enhanced self-care, problem-solving, and knowledge sharing from healthcare professionals, ultimately leading to better outcomes [41].

Limitations of this study include the moderate quality of many RCTs. Only ten studies out of the 24 RCTs had a low risk of bias. Only English-language RCTs were included, which may have excluded relevant studies published in other languages.

### What we know and what this research adds

Previous studies have shown that educational interventions and patient support play a crucial role in managing T2DM by improving glycemic control and reducing complications. This study reinforces current knowledge by performing a systematic review and meta-analysis to combine evidence on the effectiveness of educational and mentorship interventions in managing T2DM. Our research validates the beneficial impact of these interventions on glycemic control, emphasizing the potential of empowerment-based approaches to enhance diabetic self-care and clinical outcomes. The findings provide a comprehensive analysis of existing evidence, contributing valuable insights to the field of diabetes care and guiding future research and clinical practice guidelines.

### Recommendations

- Explore various health education programs that help patients, considering their preferences, requirements, and choices, which ultimately can enhance intervention tactics.
- Consider cultural and linguistic diversity when implementing interventions to ensure effectiveness across various populations globally.
- Incorporating patient education and support programs into routine clinical practice is essential. There needs to be a focus on multidisciplinary team cooperation and patient-centered care to optimize outcomes for patients with T2DM.
- Encourage diabetes centers worldwide to promote regular diabetic health education programs, especially for primary care physicians. These programs can be delivered virtually through media or conducted in person at healthcare facilities. Such initiatives can have a positive impact on patient outcomes. This recommendation is based on the local experience in Saudi Arabia, where a collaboration between the Saudi Health Council and the American Diabetic Association (ADA) exists.
- Utilize social media to expand diabetic health education, engaging healthcare professionals in sharing educational posts, hosting live streams, and creating visual content. Such strategies can improve diabetes management and patient outcomes.
- Evaluate the long-term impact of interventions on reducing diabetes-related complications, extending follow-up to assess outcomes beyond 12 months.

## CONCLUSION

Our comprehensive review and meta-analysis concluded that health education, mentorship interventions, and mentoring treatments have a significant impact on improving glycemic control in patients with T2DM. The results highlight the significance of patient support and health education through collaborative partnerships among physicians, diabetic educators, and nurses, which effectively improve glycemic control outcomes in the long term. The general trend indicates that interventions focused on empowerment, such as educational programs and patient support, can improve diabetic self-care practices and clinical outcomes.
